# Microbial dysbiosis and immune dysregulation in periodontitis and peri-implantitis

**DOI:** 10.3389/fcimb.2025.1678163

**Published:** 2026-01-07

**Authors:** Nika Mehrnia, Thomas E Van Dyke

**Affiliations:** 1 Department of Applied Oral Sciences, ADA-Forsyth Institute, Somerville, MA, United States; 2 Department of Oral Medicine, Infection and Immunity, Harvard School of Dental Medicine, Boston, MA, United States; 3 Center for Clinical and Translational Research, ADA-Forsyth Institute, Somerville, MA, United States

**Keywords:** dysbiosis, immune dysregulaiton, peri - implantitis, periodonditis, periodontal disease

## Abstract

Periodontitis and peri-implantitis are chronic inflammatory diseases which are primarily driven by excessive and dysregulated immune responses. This would result in irreversible tissue destruction around teeth and implants. Although the microbiome serves as an initiator of inflammation and leads to microbial dysbiosis, persistent and unresolved inflammation is the primary driver of tissue and bone loss. These conditions result from a dynamic interplay between the host immune response and pathogenic biofilms. Microbial dysbiosis results from a shift from a eubiotic (symbiotic) oral microbiome to a dysbiotic microbial community. This is initiated by excessive inflammation and manipulates host immunity to promote chronic inflammation. Concurrently, immune dysregulation, including imbalances in innate and adaptive immune responses that result from a failure of resolution of inflammation pathways, exacerbates tissue destruction through the overproduction of pro-inflammatory cytokines and the activation of destructive pathways, such as neutrophil-mediated degradation and osteoclast activation. This review explores the mechanisms underlying microbial dysbiosis and immune dysregulation in periodontitis and peri-implantitis, emphasizing their contribution to inflammation, bone resorption, and disease progression.

## Introduction

Periodontitis and peri-implantitis are chronic inflammatory and immune mediated diseases that are characterized by microbial dysbiosis and immune dysregulation (Kriebel et al., 2018). Both diseases result in destruction of the supporting structures around natural teeth or dental implants (alveolar bone loss). If left untreated, they can result in tooth or implant loss. The prevalence of periodontitis in dentate adults 30 years or older in US is estimated to be 42.00% (Eke et al., 2018). In comparison, the prevalence of peri-implantitis is estimated 19.53%at the patient level and 12.53%at the implant level (Diaz et al., 2022).

Bacteria are the initial etiologic factor in both periodontitis and peri-implantitis, which results in inflammation (gingivitis) that if severe and long-lasting leads to microbiome dysbiosis. Microbial dysbiosis is an imbalance in the composition and diversity of the oral microbiome that perpetuates and exacerbates the development and progression of both periodontitis and peri-implantitis. This shift from a healthy, eubiotic microbial community to a dysbiotic, pathogenic community, leads to changes in host–microbe crosstalk, triggering excessive inflammatory responses and bone loss (Hajishengallis, 2014; Scannapieco and Dongari-Bagtzoglou, 2021; Wei et al., 2024). Although microbial dysbiosis plays a crucial role in disease etiology, immune dysregulation is the key contributor to pathogenesis and tissue destruction. The biofilm triggers an excessive immune response that is initially protective but becomes maladaptive as it progresses toward chronic inflammation. According to the literature, the disease is not caused solely by microbial dysbiosis or the host immune response alone; rather, it results from a combination of both. However, the precise role and relative contribution of these factors each remain poorly understood (Sedghi et al., 2021; Van Dyke et al., 2020).

The classical understanding of periodontitis as a disease caused by specific pathogenic bacteria has evolved and evidence suggests that periodontitis and peri-implantitis are not simply infections caused by a few select microorganisms but are instead manifestations of a complex interplay between the subgingival microbiota and the host immune response. While bacteria are indeed essential for disease initiation, as demonstrated by the reversal of gingivitis following plaque removal and bone loss prevention in germ-free animals, the onset of periodontitis is now understood to involve microbial dysbiosis rather than the acquisition of new pathogens. Dysbiosis is driven by changes in the local environment like the host’s inflammatory response (Curtis et al., 2020). Studies show that actively resolving inflammation can reverse dysbiosis and restore tissue integrity, highlighting the bidirectional relationship between host and microbiota (Hasturk et al., 2006; Van Dyke and Serhan, 2003).

Current evidence strongly suggests periodontitis is not merely an infectious condition caused by specific pathogens but rather an inflammatory disease driven by dysregulated host immune responses to microbial dysbiosis. The persistent inflammation and subsequent tissue destruction are primarily immune-mediated, emphasizing the critical role of inflammation resolution pathways in disease management.

This review aims to provide an overview of the pathogenic mechanisms underlying periodontitis and peri-implantitis, with a specific focus on the role of immune dysregulation and the resulting microbial dysbiosis.

## Periodontitis

### Microbial dysbiosis in periodontitis

The transition from a healthy, eubiotic oral microbiome to a dysbiotic, disease-provoking microbial community is the main feature in the initiation of pathogenesis of periodontitis (Hajishengallis, 2015). Although it is initially triggered by microbial biofilms, the transition to dysbiosis in periodontitis is largely facilitated by the host’s inflammatory environment. This inflammation driven dysbiosis selects for pathogenic microbial species capable of thriving under conditions of chronic inflammation, thereby perpetuating immune dysregulation rather than causing direct infection.

**Figure 1:**
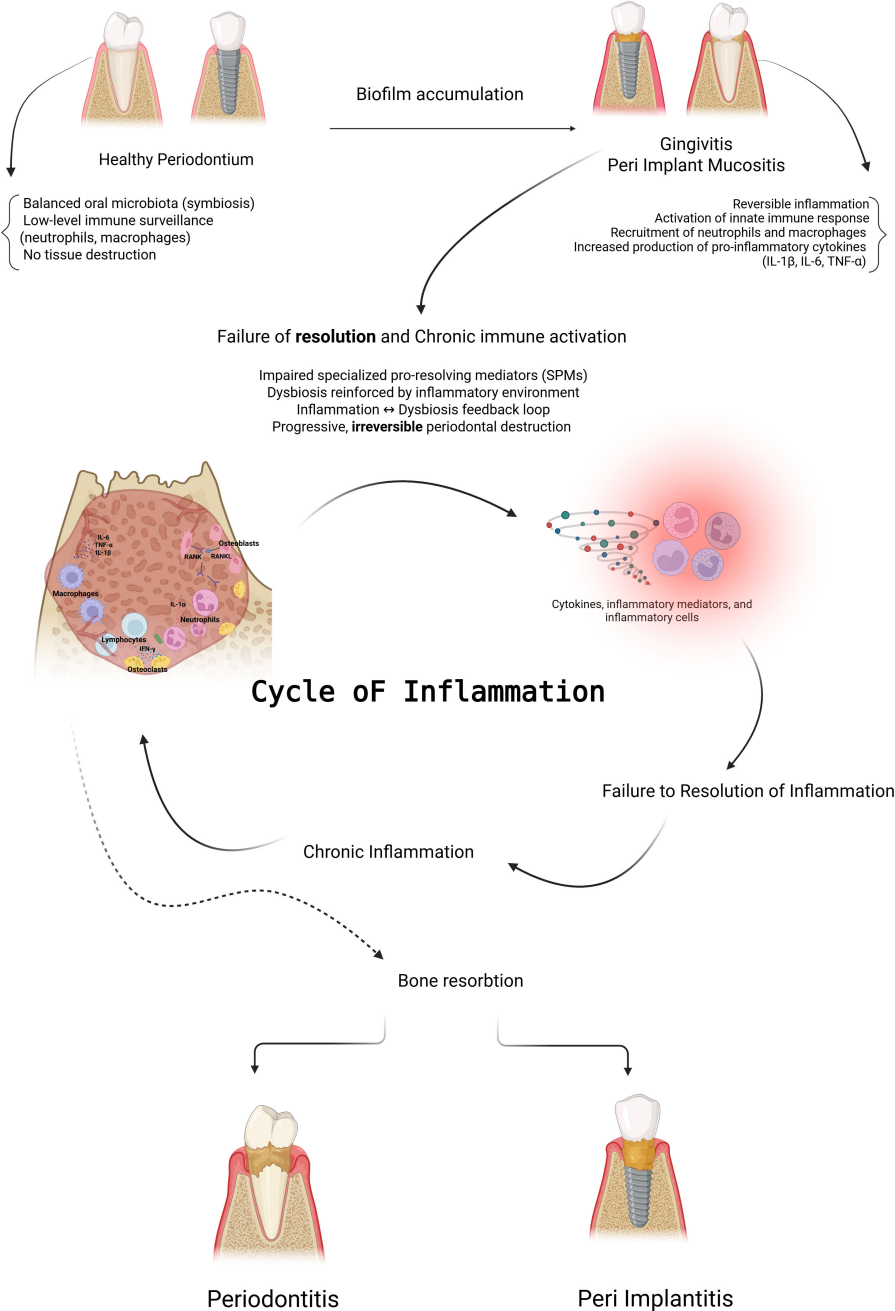
Cycle of inflammation in periodontal and peri-implant tissues. Under healthy conditions (left), a balanced oral microbiome and low level immune response maintain soft tissue and bone homeostasis around both teeth and implants. Biofilm accumulation triggers gingivitis or peri-implant mucositis, characterized by reversible activation of the innate immune response, neutrophil and macrophage recruitment, and elevated proinflammatory cytokines (IL1β, IL6, TNFα). In the middle, the diagram illustrates the cellular and molecular events occurring within inflamed periodontal/peri-implant tissues. This process reflects the failure of resolution, which happens when SPMs fail to restore balance and a dysbiosis inflammation feedback loop develops. This would lead to chronic immune activation and irreversible tissue destruction. (right) (Mehrnia, 2025a).

In a healthy state, the oral microbiome maintains a eubiotic relationship with the host in a stable and balanced ecosystem. This balance supports periodontal health by regulating microbial diversity and limiting pathogenic overgrowth. The gingival microbiome associated with periodontal health remains in dynamic equilibrium with the host, supporting oral health. However, this balance is disrupted during the progression to gingivitis and leads to dysbiosis in the progression to periodontitis. While gingivitis is a reversible inflammatory condition that reflects a state of microbial/host homeostasis, chronic and uncontrolled inflammation drives a shift in the microbial community, that results in the irreversible destruction of hard and soft tissues (**Figure 1**). The key unresolved question is why and how healthy gingival tissue progresses to gingivitis and, ultimately, periodontitis. For many years the main focus was on the disease associated bacteria, which constitute a minor part of the subgingival microbiome in health and increase significantly as periodontal pockets form, and periodontitis develops. This microbial shift is accompanied by an increase in anaerobic gram-negative bacteria, leading to a pathogenic synergy that exacerbates host tissue destruction (Hajishengallis and Lamont, 2012; Van Dyke et al., 2020).

Based on these facts, it was supposed that there are some specific bacteria that are the main cause of the disease, and if they are present in the microbiome around the tooth, the healthy periodontium would develop gingivitis and later periodontitis. This was the main idea for the keystone pathogen hypothesis. It suggested that certain low-abundance microbial species play an important role in disease progression by orchestrating shifts in the microbial community and inducing host immune dysregulation, and in the context of periodontitis, *Porphyromonas gingivalis* is a suggested keystone pathogen. Despite low abundance within the oral microbiome, *P. gingivalis* exerts a significant influence on microbial community structure and host immunity (Hajishengallis et al., 2012; Sedghi et al., 2021).

Based on this idea, it was suggested that the microbiome associated with the periodontitis is an overgrowth of select commensals termed pathobionts (Curtis et al., 2020). Pathobionts are opportunistic microbes that are typically part of the host microbiome and are generally harmless. However, under adverse conditions, they can shift their phenotype to a pathogenic state, that amplifies inflammation and leads to disease progression (Chandra et al., 2021).

The literature supports the fact that the main etiology for gingivitis is bacterial challenge, and removal of the plaque results in reestablishment of healthy conditions (Curtis et al., 2020; Löe et al., 1965). If the gingivitis does not resolve, in many cases it will progress toward periodontitis. This process is accompanied by progressive dysbiosis; however, the exact mechanism is still not clear (Curtis et al., 2020).

Subsequent research has shown that disease development cannot be explained simply by the presence of a few specific bacterial species, even though the microbiome composition does change. The original keystone-pathogen hypothesis which suggests that a single microbe could dominate and trigger disease, has given way to a broader view that the entire microbial community must be considered. Once this community becomes dysbiotic, it can push the periodontium toward disease; however, that shift involves the collective community, not just one bacterial species. Still, this perspective does not fully answer the lingering question of “who is responsible?” because many microbes found in dysbiotic sites also appear in periodontally healthy individuals and even if this is the main reason, why does this change in the microbiome happen. To resolve this dilemma, we may need to shift our focus to the other side of the battlefield, the host periodontium itself.

Increasing inflammation in the host environment selects for specific bacterial species based on their growth requirements. As inflammation increases beyond normal levels, it fosters conditions that preferentially support the proliferation of gram-negative and proteolytic bacterial populations. Once established, these bacteria actively amplify and perpetuate the inflammatory response, further enriching their niche with tissue breakdown products. Consequently, a self-sustaining cycle emerges, wherein inflammation and bacterial overgrowth reinforce each other, this enables these selected bacterial groups to maintain their selective dominance within the inflamed environment (Bartold and Van Dyke, 2019; Van Dyke et al., 2025).

While the inflammatory response is a protective mechanism against pathogens, it can have adverse consequences when excessive or chronic (Pan et al., 2019a). Prolonged inflammation can result in tissue destruction through mechanisms such as neutrophil mediated degradation and the action of matrix metalloproteinases (Sirisereephap et al., 2022). The dysbiotic microbiome recruits immune cells to the periodontium and triggers the release of inflammatory cytokines, including IL-1, IL-6, and TNF-α (Meuric et al., 2017). These cytokines stimulate osteoclast activity, ultimately leading to bone loss and contributing to the progression of periodontitis (Hajishengallis and Lamont, 2021).

So maybe we have found an answer to our initial question and inflammation is the underlying driver of the disease. As mentioned earlier, prolonged and uncontrolled inflammation is the issue and not surprisingly many other chronic disorders share the same underlying pathology. In fact, persistent, unresolved inflammation plays a central role in these disease such as type 2 diabetes, cardiovascular disease, and rheumatoid arthritis (Araújo et al., 2015; Kampits et al., 2016; Preshaw and Bissett, 2019). But now this opens a new question, why and how the inflammation, which is a response that begins as a protective mechanism lose its regulatory mission, turning into a destructive force that causes more damage than the external invaders—the dysbiotic bacterial community?

To address this question, we need to understand what defines the endpoint of inflammation and what it truly means for inflammation to be uncontrolled. Is there a biological mechanism responsible for resolving inflammation and restoring tissue homeostasis?

For many years, it was believed that homeostasis simply returned once inflammation subsided, making resolution a passive consequence of an inflammation free state. We now know, however, that resolving inflammation is an active, highly regulated process driven by specialized pro-resolving mediators (SPMs). These molecules are the principal agents of resolution: they orchestrate the shutdown of inflammatory pathways and restore tissue balance. Although SPMs are produced endogenously, their levels decline rapidly with age, which may partly explain why chronic inflammatory diseases become more prevalent later in life. Unlike anti-inflammatory medications such as NSAIDs, which block inflammation, SPMs “shift” the inflammation status toward resolution. Under these conditions, tissue regeneration is promoted, and homeostasis is restored. Moreover, SPMs modify the microbiome’s response to inflammation, an effect that NSAIDs cannot achieve (Van Dyke, 2017).

In contrast to the earlier belief that a single “superstar” pathogen drives periodontal diseases, the story involves several actors—a dysbiotic microbiome, dysregulated inflammation, and impaired resolution pathways. Yet a central mystery persists: why does a once-healthy periodontium begin to deteriorate? Why does the process remain limited to gingivitis in some individuals but progress to periodontitis in others? Who enters the stage first, and in what sequence do the other players appear—or do they all act simultaneously? These questions remain unanswered and require further research. Thus, we need to focus on the different aspects of periodontal diseases pathogenesis.

### Role of innate and adaptive immunity in periodontitis

Although the innate and adaptive immune systems are essential for periodontal defense, their sustained activation becomes maladaptive, driving chronic inflammation and tissue destruction characteristic of periodontitis. This pathological immune activity, rather than direct actions of bacteria, leads to irreversible bone and tissue loss.

The innate immune system serves as the first line of defense against periodontal pathogens. It comprises various immune cells, including macrophages, neutrophils, and dendritic cells, which recognize and respond to microbial invaders through pattern recognition receptors (PRRs), such as Toll-like receptors (TLRs) (Cekici et al., 2014; Chukkapalli et al., 2017). TLRs are part of the host innate immune response that have a pivotal role in recognizing the pathogens and activating the innate immune system (Song et al., 2017). The TLRs recognize pathogen-associated molecular patterns (PAMPs) and recruit adaptor proteins to initiate signaling pathways. TLR uses MyD88-dependent and MyD88-independent (TRIF-dependent) pathways. The MyD88-dependent pathway activates NF-kB and AP-1, while the TRIF-dependent pathway leads to IRF3 activation, promoting the production of pro-inflammatory cytokines and interferons (IFN-α and IFN-β), which regulate host innate immunity. The activation of Toll-like receptors (TLRs) stimulates the release of pro-inflammatory cytokines and chemokines, attracting more immune cells to the infection site and intensifying the inflammatory response. Although this initial reaction is protective, failure to regulate it can result in chronic inflammation and tissue damage (Song et al., 2017).

On the other hand, the adaptive immune system provides a more targeted and durable response, involving T and B lymphocytes. These cells are critical for immunological memory and antibody production. In periodontitis, CD4+ T-helper cells, particularly the Th1 and Th17 subsets, are essential to drive inflammatory responses and contribute to bone resorption (Wang et al., 2021). The interaction between innate and adaptive immunity is essential, as antigen-presenting cells (APCs), such as dendritic cells, bridge the two systems by activating T cells (naïve CD4+ and CD8+ T cells) (Chudnovskiy et al., 2019). This interplay ensures a pathogen-specific immune response which is designed to address the various microbial challenges present in the periodontal biofilm. Th17 and Treg cells have an important dual influence over periodontal inflammation and tissue destruction. Th17 cells, driven by cytokines such as IL-6 and TGF-β, expand early during disease progression and secrete IL-17A/F, which enhances neutrophil recruitment, stimulates local production of IL-1β, TNF-α, and IL-6, and strongly promotes osteoclastogenesis through RANKL upregulation, thereby accelerating alveolar bone loss. In contrast, regulatory T cells (Tregs) maintain tissue homeostasis by limiting excessive immune activation via IL-10, TGF-β, CTLA-4, and contact-dependent suppression. In health, Tregs effectively counterbalance Th17-mediated inflammation, restraining soft-tissue damage and inhibiting osteoclast differentiation. However, chronic periodontal inflammation disrupts this balance: Tregs become phenotypically unstable, downregulate Foxp3, and may even acquire Th17-like features, including IL-17A expression, which diminishes their suppressive capacity and further amplifies bone-destructive pathways (Alvarez et al., 2020; Baima et al., 2025). Thus, the Th17/Treg axis functions as a critical immunological rheostat in periodontitis, where a shift toward Th17 dominance and loss of Treg stability actively drives the transition from controlled inflammation to irreversible periodontal breakdown.

**Figure 2:**
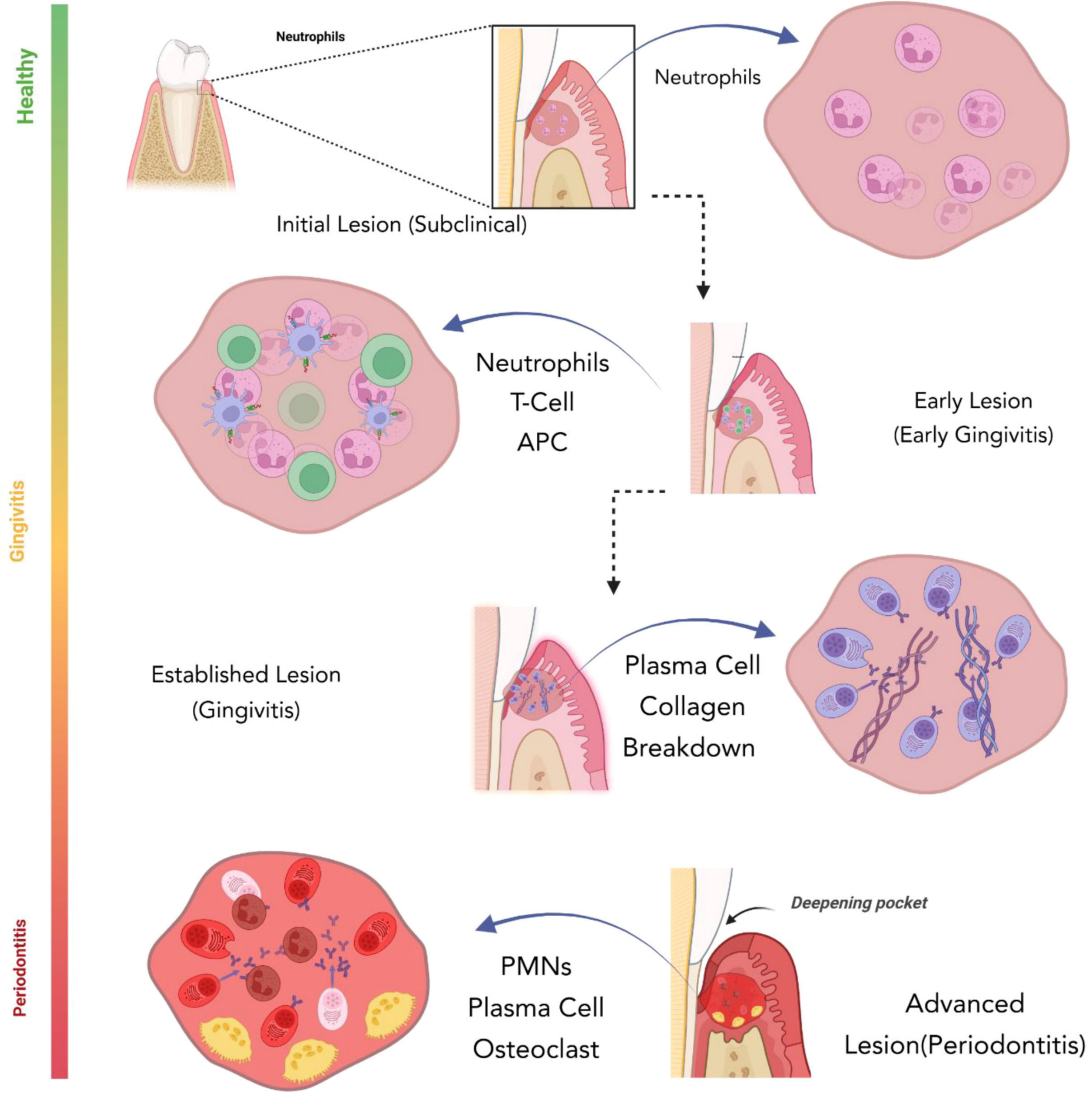
Immune response across periodontal lesion stages. The figure shows how innate and adaptive immune responses evolve from health to gingivitis and periodontitis. The initial lesion is dominated by neutrophil-driven innate immunity. In the early lesion, APCs and T cells signal the onset of adaptive responses. The established lesion features plasma cell dominance and collagen breakdown. In the advanced lesion, pocket formation, heavy neutrophil infiltration, plasma cells, and osteoclast activity drive irreversible tissue and bone destruction (Mehrnia, 2025b). SPMs orchestrate the resolution phase by mechanisms such as inhibiting neutrophil infiltration, which is crucial for preventing neutrophil-mediated tissue destruction. Lipoxins (e.g., lipoxin A_4_ and lipoxin B_4_) limit neutrophil recruitment, chemotaxis, and adhesion, while resolvins are highly potent inhibitors of neutrophil infiltration and transmigration. These molecules coordinate the resolution of inflammation. Again, it is worth mentioning that the SPMs do not block inflammation like NSAIDs but rather return tissues to a state of homeostasis. SPM-mediated pro-resolution includes other actions like prompting macrophages to clear apoptotic cells without triggering further inflammation (Serhan et al., 2008).

The gingivitis stages described by Page and Schroeder (Page and Schroeder, 1976) align with phases of innate and adaptive immunity. In the first stage (the initial lesion) polymorphonuclear neutrophils (PMNs) predominate indicating active innate immunity (**Figure 2**). This stage is often called *subclinical gingivitis* because virtually all healthy individuals have some degree of inflammation and being entirely inflammation-free is uncommon in clinical practice. Importantly, low-level inflammation is not fundamentally harmful, and a resident microbiome is almost always present. At this point, both the microbiome and an early inflammatory response coexist, yet the patient is still classified as clinically healthy and may remain in this state indefinitely. Whether the response resolves or progresses to chronic inflammation depends on its intensity and character.

The early lesion follows, marked by visible clinical signs of gingivitis and dominated by lymphocytes (primarily T cells). If the disease advances, an established lesion develops, characterized by plasma-cell dominance. Although innate and adaptive immune mechanisms operate throughout these three stages, all are still considered gingivitis, reversible inflammation. If the inflammation continues, the process enters the advanced lesion stage. Here, the lesion involves the alveolar bone, periodontitis begins, and the disease becomes chronic. Neutrophils dominate the epithelium, and tissue damage is driven largely by the host inflammatory response rather than the biofilm itself.

The transition from stage 3 to stage 4 is a critical checkpoint: the shift from acute to chronic inflammation makes damage irreversible. While both biofilm and host response contribute to disease progression, controlling and resolving inflammation is pivotal. Notably, in the initial stage, biofilm and an acute inflammatory response coexist in individuals who remain clinically healthy. This demonstrates that their mere presence is not inherently pathogenic. Even if early stages progress, stages 2 and 3 remain reversible so proper regulation can prevent progression to the advanced lesion.

## The dysregulation of immune pathways and cytokine networks

The defining feature of periodontitis is the dysregulated immune response, the excessive and persistent production of pro-inflammatory cytokines and the failure of resolution of inflammation pathways, which not only perpetuates inflammation but directly mediates tissue and bone destruction independent of ongoing microbial stimulation. One of the key features of immune dysregulation in periodontitis is the imbalance between pro-inflammatory and anti-inflammatory cytokines. Pro-inflammatory cytokines, such as TNF- α, IL-1 β, and IL-6, are produced in response to bacterial infection and are crucial for initiating the inflammatory response (Pan et al., 2019b). However, excessive production of these cytokines can cause tissue destruction and bone resorption, as they promote osteoclastogenesis and the recruitment of inflammatory cells to periodontal tissues (Cekici et al., 2014). The key point is that proinflammatory cytokines aren’t harmful on their own, but when they become uncontrolled or there is a failure to engage the proresolution pathway, they drive unwanted tissue destruction. In the acute inflammatory phase, proinflammatory mediators initiate- a protective response, while SPMs are simultaneously produced to turn that response off at the appropriate time. SPMs promote resolution through multiple mechanisms like limiting neutrophil recruitment and reprogramming macrophages from a proinflammatory to an anti-inflammatory- phenotype. By doing so, SPMs make the inflammation self limiting- and drive effective clearance (Basil and Levy, 2016). If this balance is disrupted, the proinflammatory signals persist and will perpetuate inflammation and recruit more cells and cytokines in a vicious, tissue damaging- cycle. This interplay underlines how essential timely control by proresolution mediators is: when both arms work together, inflammation remains- protective; if not, the resulting tissue injury can exceed the damage posed by the original pathogen or pathobiont (Bassani et al., 2023).

## Mechanisms of tissue destruction mediated by the immune system

The destruction observed in periodontitis primarily arises from dysregulated immune pathways, where pro-inflammatory mediators and immune cell-derived enzymes, rather than bacteria themselves, cause direct tissue breakdown. The immune system, intended to protect periodontal tissues, paradoxically becomes the main driver of periodontal destruction.

One of the primary mechanisms of tissue destruction in periodontitis is the overproduction of pro-inflammatory cytokines. Cytokines such as IL-1 βand TNFα are released in response to periodontal pathogens, leading to an inflammatory cascade that promotes tissue breakdown (Ertugrul et al., 2013). These cytokines stimulate the activity of osteoclasts, the cells responsible for bone resorption, thus contributing to the alveolar bone loss of periodontitis. Furthermore, the imbalance between pro-inflammatory mediators and their inhibitors exacerbates tissue destruction, as the excessive inflammatory response overwhelms the body’s ability to repair and regenerate tissues (Ertugrul et al., 2013).

Another critical factor in tissue destruction is the role of neutrophils. In periodontitis, neutrophils are recruited to the site of infection and become activated, leading to the release of reactive oxygen species (ROS) and various tissue-damaging enzymes, such as collagenase and elastase (Zaric et al., 2010). IL-17 is another pro inflammatory cytokine produced by Th17 (Lin et al., 2014). In periodontitis, IL-17 signaling promotes the expression of matrix metalloproteinases (MMPs) and receptor activator of nuclear factor kappa-B ligand (RANKL), both of which play crucial roles in bone resorption and connective tissue degradation (Beklen et al., 2007). Dysregulation of IL-17 and its associated pathways can therefore lead to increased tissue destruction and exacerbate the progression of periodontitis.

Developmental endothelial locus-1 (DEL-1) is secreted by endothelial cells and has the capacity to regulate immune functions and play a role in inflammatory processes (Hajishengallis and Chavakis, 2019). It can bind to LFA-integrin on the neutrophils surface, which blocks neutrophil adhesion to ICAM-1 and inhibits neutrophil extravasation to the tissue. This is important as neutrophils play a key role in tissue destruction and inflammatory bone loss. In addition to this, DEL-1 directly antagonizes IL-17 mediated inflammation, whereas IL-17 suppresses DEL-1 expression and thereby promotes excessive neutrophil recruitment. During the resolution of inflammatory response, DEL-1 promotes efferocytosis, enhances apoptotic cell clearance, and drives pro-resolving macrophage polarization. DEL-1 is upregulated by resolvin D1 (Maekawa et al., 2015). In addition, by suppressing the MMP and RANKL expression, it prevents bone loss. Mechanistically, the protein’s RGD (Arg–Gly–Asp) motif allows engagement with integrins involved in both initiation and resolution of inflammation. Interaction with αvβ3 integrin enhances efferocytosis and promotes pro-resolving macrophage programming, while also inhibiting αvβ3-dependent activation of pro-MMP2, a major mediator of extracellular matrix degradation and a key factor in periodontal tissue destruction. Beyond the periodontium, experimental studies have demonstrated therapeutic benefits of DEL-1 in cardiovascular and metabolic diseases (CVMDs) and in hypertension-induced cardiovascular remodeling (Failer et al., 2022; Zhao et al., 2022).

Preclinical evidence also indicates that DEL-1 acts as an endogenous regulator of neuroinflammation within the central nervous system. It restricts leukocyte adhesion, preserves blood–brain barrier integrity, and limits IL-17–driven inflammatory cascades. Reduced DEL-1 levels correlate with heightened inflammation and disease severity, whereas systemic administration of a DEL-1–Fc fusion protein ameliorates disease relapse severity. Collectively, these findings highlight DEL-1’s therapeutic potential in maintaining CNS immune homeostasis and mitigating pathogenic inflammatory responses with broad therapeutic potential across inflammatory conditions (Choi et al., 2015). Nonetheless, it’s important to remember that regulation of DEL-1 expression by SPMs is upstream of these activities and treatment with SPMs have many of the same impact suggesting that SPM actions may be affected in large part through DEL-1 pathways.

As noted earlier, the hallmark consequence of periodontitis is alveolar bone resorption and it results primarily from dysregulated immune pathways rather than from the mere presence of biofilm or inflammation itself. This underscores the importance of controlling and properly regulating the inflammatory response. SPMs are of particular interest because they both prevent further tissue destruction and promote repair once deemed irreversible.

In a rabbit model of ligature-induced periodontitis with *P. gingivalis*, topical application of Resolvin E1 resolved inflammation and produced measurable bone gain. This highlights SPMs’ ability to regenerate bone while dampening destructive inflammation (Hasturk et al., 2007). Similarly, in a rat ligature‐induced periodontitis model, Lee et al. (2016) showed that Resolvin E1 (RvE1) both prevents and reverses periodontal tissue destruction and associated microbial dysbiosis. In prevention experiments, RvE1 halted alveolar bone loss, reduced osteoclast density, and limited inflammatory cell infiltration. In treatment experiments, it regenerated lost bone and normalized gingival inflammatory gene expression profiles as assessed by RNA-seq. 16S rDNA sequencing showed that local inflammation control reshapes the subgingival microbiota toward a healthier composition (Lee et al., 2016). These are interesting findings as the SPMs do not have direct antimicrobial actions and their impact on the microbiome is a manifestation of the immune system (Hasturk et al., 2006).

Lee et al. (2021) further investigated how disruptions in SPM pathways relate to shifts in the subgingival microbiome in periodontitis. They analyzed gingival tissue and plaque from healthy and periodontitis subjects using metabololipidomic profiling of SPMs, qRT-PCR of SPM receptor genes, and 16S rRNA sequencing. They revealed the association between lipid mediator levels and microbiome composition. Notably, correlations identified key lipid mediators and bacterial species such as *Corynebacterium durum* linked with 5(S)6(R)-DiHETE in health versus disease and *Anaeroglobus geminatus* in treatment response. This highlights the lipid-driven resolution signals as critical modulators of periodontal microbial ecology (Lee et al., 2021).

The role of SPMs and their downstream effects have been studied in clinical trials as well. In a randomized clinical trial, Hasturk et al. (2021) reported the use of an oral rinse containing a stable lipoxin A4 analog (BLXA4) in adults with gingival inflammation. Over 28 days, compared to placebo and control groups, BLXA4 produced greater reductions in gingival inflammation and pocket depth, and it significantly increased systemic levels of specialized proresolving lipid mediators. This data suggests the SPMs as a safe, effective host modulation strategy for periodontal diseases (Hasturk et al., 2021).

## Peri-implantitis

### Microbial dysbiosis in peri-implantitis

Studies have shown that the microbial communities associated with peri-implant biofilms are often dominated by specific pathogens that are also implicated in periodontal diseases. For instance, *P. gingivalis* and *Fusobacterium nucleatum* are frequently identified in biofilms from peri-implantitis sites (Blank et al., 2021; Ghensi et al., 2020; Li et al., 2023).

The evidence does not demonstrate a consistent specific microbial profile for peri-implantitis. While certain bacterial species, such as *P. gingivalis*, *F. nucleatum*, *Tannerella forsythia*, and *Aggregatibacter actinomycetemcomitans*, are frequently associated with peri-implantitis, their presence is not exclusive to the condition and can also be found in periodontitis and even in healthy sites to varying extents. Additionally, *Staphylococcus epidermidis*, a species less commonly associated with natural teeth, is often detected in peri-implantitis cases. Interestingly, *S. epidermidis* has been found to colonize peri-implant tissues rather than implant surfaces, suggesting a potential role in infections involving free-floating (planktonic) bacteria. Given its established involvement in planktonic infections of biomaterials, *S. epidermidis* might contribute to a distinct form of peri-implantitis, differing from the conventional type associated with red and orange complex bacteria. Additionally, studies reveal considerable variability in microbial composition between individuals and across different studies, with no single species consistently characterizing peri-implantitis. This heterogeneity highlights the complexity of peri-implant biofilms and the influence of host and environmental factors on microbial communities (Carvalho et al., 2023; Sahrmann et al., 2020).

### Immune responses in peri-implantitis

As in periodontitis, the host immune system reacts to peri-implant biofilms by recognizing microbial components through receptors such as Toll-like receptors (TLRs). This recognition initiates a cascade of inflammatory responses, leading to the release of cytokines like IL-1β, IL-6, and TNFα, which recruit immune cells such as neutrophils and macrophages to the site. These cells amplify the response by producing more inflammatory mediators, enzymes, and chemokines. While this response is meant to control the microbial threat, its dysregulation often leads to persistent inflammation, tissue damage, and ultimately bone loss around implants (Giro et al., 2021; Kensara et al., 2021).

Although peri-implantitis and periodontitis share similarities as biofilm-induced inflammatory diseases, they exhibit differences in immune response intensity and cellular involvement. Peri-implantitis generally presents a more exaggerated inflammatory response with increased levels of cytokines like IL-17 and greater recruitment of neutrophils. This heightened immune activity reflects a more aggressive environment compared to periodontitis. Additionally, peri-implantitis is marked by an imbalance between T helper 17 (Th17) cells and regulatory T cells (Tregs), which exacerbates inflammation. Conversely, periodontitis often shows a relatively more balanced Th17/Treg dynamic, though it still involves chronic inflammation. The unique structural and material properties of implants may also contribute to the differences in immune response (Giro et al., 2021).

### Role of inflammation and bone resorption in peri-implantitis

Inflammation plays a central role in peri-implantitis by driving tissue and bone destruction. Pro-inflammatory cytokines such as IL-6 and TNF -α stimulate osteoclast activity through the RANK/RANKL pathway, accelerating bone resorption. The persistent inflammation, fueled by a dysbiotic biofilm, undermines the peri-implant tissues’ ability to repair, leading to progressive bone loss and compromised implant stability. This destructive process differentiates peri-implantitis as a more aggressive and complex condition compared to other inflammatory oral diseases (Giro et al., 2021; Kensara et al., 2021).

### Potential role of foreign body reactions in peri-implantitis

Foreign body reactions play a modulatory, but not primary, role in the pathogenesis of peri-implantitis and the role of these reactions in peri-implantitis is increasingly recognized alongside biofilm-mediated inflammation. From osteoimmunology point of view, osseointegration itself is a controlled foreign body equilibrium, in which the immune system and macrophage polarization pathways continuously “shield off” the titanium surface to maintain functional integration (Albrektsson et al., 2022).

When this equilibrium is disturbed, chronic inflammation may transition toward a maladaptive foreign body response characterized by persistent M1-type activation, impaired resolution, and susceptibility to marginal bone loss. A growing body of evidence shows that titanium particles and ions accumulate in peri-implant tissues and the concentration is significantly increased in peri-implantitis site (Chen et al., 2023). These particles have the potential to stimulate macrophages and neutrophils, enhancing secretion of TNF-α, IL-1β, IL-6, and reactive oxygen species, while inducing cellular degeneration and genotoxic effects (Noronha Oliveira et al., 2018). Histologic studies demonstrate that foreign body giant cells, chronic inflammatory infiltrates, and impaired immune clearance contribute to a microenvironment where titanium debris, dysbiotic biofilm, and host response collectively promote progressive osteolysis. Recent insights further support that foreign body stimulation can disrupt the immune system and the subsequent immune dysregulation leads to an exaggerated response, altered T-cell balance, and RANKL-mediated osteoclastogenesis which would accelerate the destruction of the tissue in peri-implantitis (Huang et al., 2024).

## Host genetic factors

Periodontitis and peri-implantitis are multifactorial diseases and result from the interaction of microbial dysbiosis, environmental exposures, and host-related factors (Modafferi et al., 2025). Beyond the biofilm challenge, genetic susceptibility influences the onset, severity, and progression of periodontitis. Twin studies demonstrate a clear genetic contribution, with monozygotic twins exhibiting more than twice the risk of early-onset periodontitis compared with dizygotic twins, and heritability for chronic periodontitis estimated at approximately 50%. Several genetic variants associated with periodontal susceptibility involve regulators of innate immune responses and mucosal integrity, including *SIGLEC5* and *DEFA1A3* (Richter and Schaefer, 2025). The *CDKN2B-AS1* (ANRIL) locus on chromosome 9p21.3, a highly pleiotropic region previously linked to coronary artery disease, diabetes, stroke, and Alzheimer’s disease, has also been associated with periodontitis, likely through its regulatory effects on gene expression and inflammatory pathways (Loos and Van Dyke, 2020). Recent large-scale genomic data from the FinnGen study further identified several independent risk loci for periodontitis, including variants near *FST*, *ARL15*, *MFHAS1*, and *DEFB130A*, as well as strong associations within the HLA class II region, highlighting the central role of immune-regulatory genetic variation in disease susceptibility (Salminen et al., 2025). These genetic variants influence cytokine signaling, neutrophil activity, tissue repair mechanisms, and epithelial barrier stability. Consequently, individuals with similar microbial exposures may exhibit widely different clinical outcomes, underscoring the importance of polygenic risk architecture in periodontal diseases pathobiology.

## Conclusion

### Interplay between microbial dysbiosis and immune dysregulation

Although microbial dysbiosis is associated with the amplification and perpetuation of the inflammatory cascade, the hallmark of periodontitis and peri-implantitis progression is a sustained dysregulation of immune responses. Rather than the presence of pathogens, failure to resolve inflammation is what drives ongoing tissue destruction.

The microbial component is considered the initial starting point for these diseases, although its role is not fully understood. All cases of the disease have a microbial component present in the oral cavity. However, the presence of the microbiome does not necessarily induce periodontal diseases, as microbial components thought to be pathogenic can also be detected in healthy individuals. On the other hand, the role of the host immune system is critical. The microbial component acts as a trigger for the destructive process, but it is the host immune system that causes the actual destruction of periodontal and peri-implant tissue. While the exact mechanisms and interplay between the microbiome and the host immune system remain unclear, it is evident that tissue destruction primarily results from the host immune response. The inability of the immune system to eliminate the infection, combined with the uncontrolled activity of the immune system, leads to progressive tissue damage.

The reason behind this phenomenon is still not fully understood. However, if the body’s immune system cannot eliminate the infection and resolve the initial inflammation caused by the microbial component, the inflammation becomes chronic, resulting in tissue destruction through mechanisms such as neutrophil-mediated damage. A key factor in this process is the failure of the immune system to resolve inflammation. Inflammation is typically a protective response, but it may lose its protective nature when it becomes chronic and uncontrolled, which leads to further damage. Contrary to the old belief that inflammation resolution occurs passively once inflammation subsides, it is now clear that resolution is an active process, and SPMs play a crucial role in this process.

The resolution of inflammation is an intriguing area of study, as SPMs not only resolve inflammation but also heal diseased tissue and promote regeneration. The exact mechanisms of periodontal and peri-implant disease initiation and progression are coming into focus. It can be concluded that the failure to resolve inflammation is a critical factor. While questions still exist, SPMs have shown a promising role in restoring homeostasis and promoting bone regeneration, offering hope for new therapeutic approaches in managing periodontitis and peri-implantitis.

## References

[B1] Albrektsson T. Tengvall P. Amengual L. Coli P. Kotsakis G. A. Cochran D. (2022). Osteoimmune regulation underlies oral implant osseointegration and its perturbation. Front. Immunol. 13. doi: 10.3389/fimmu.2022.1056914, PMID: 36761175 PMC9902598

[B2] Alvarez C. Suliman S. Almarhoumi R. Vega M. E. Rojas C. Monasterio G. . (2020). Regulatory T cell phenotype and anti-osteoclastogenic function in experimental periodontitis. Sci. Rep. 10, 19018. doi: 10.1038/s41598-020-76038-w, PMID: 33149125 PMC7642388

[B3] Araújo V. M. A. Melo I. M. Lima V. (2015). Relationship between periodontitis and rheumatoid arthritis: review of the literature. Mediators Inflamm. 2015, 259074. doi: 10.1155/2015/259074, PMID: 26347200 PMC4539505

[B4] Baima G. Arce M. Romandini M. Van Dyke T. (2025). Inflammatory and immunological basis of periodontal diseases. J. Periodontal Res. doi: 10.1111/jre.70040, PMID: 41065279

[B5] Bartold P. M. Van Dyke T. E. (2019). An appraisal of the role of specific bacteria in the initial pathogenesis of periodontitis. J. Clin. Periodontol. 46, 6–11. doi: 10.1111/jcpe.13046, PMID: 30556922 PMC6357965

[B6] Basil M. C. Levy B. D. (2016). Specialized pro-resolving mediators: endogenous regulators of infection and inflammation. Nat. Rev. Immunol. 16, 51–67. doi: 10.1038/nri.2015.4, PMID: 26688348 PMC5242505

[B7] Bassani B. Cucchiara M. Butera A. Kayali O. Chiesa A. Palano M. T. . (2023). Neutrophils’ Contribution to periodontitis and periodontitis-associated cardiovascular diseases. Int. J. Mol. Sci. 24, 15370. doi: 10.3390/ijms242015370, PMID: 37895050 PMC10607037

[B8] Beklen A. Ainola M. Hukkanen M. Gürgan C. Sorsa T. Konttinen Y. T. (2007). MMPs, IL-1, and TNF are regulated by IL-17 in periodontitis. J. Dent. Res. 86, 347–351. doi: 10.1177/154405910708600409, PMID: 17384030

[B9] Blank E. Grischke J. Winkel A. Eberhard J. Kommerein N. Doll K. . (2021). Evaluation of biofilm colonization on multi-part dental implants in a rat model. BMC Oral. Health 21, 313. doi: 10.1186/s12903-021-01665-2, PMID: 34144677 PMC8212458

[B10] Carvalho É.B.S. Romandini M. Sadilina S. Sant’Ana A. C. P. Sanz M. (2023). Microbiota associated with peri-implantitis-A systematic review with meta-analyses. Clin. Oral. Implants Res. 34, 1176–1187. doi: 10.1111/clr.14153, PMID: 37523470

[B11] Cekici A. Kantarci A. Hasturk H. Van Dyke T. E. (2014). Inflammatory and immune pathways in the pathogenesis of periodontal disease. Periodontol 64, 57–80. doi: 10.1111/prd.12002, PMID: 24320956 PMC4500791

[B12] Chandra H. Sharma K. K. Tuovinen O. H. Sun X. Shukla P. (2021). Pathobionts: mechanisms of survival, expansion, and interaction with host with a focus on Clostridioides difficile. Gut Microbes 13, 1979882. doi: 10.1080/19490976.2021.1979882, PMID: 34724858 PMC8565823

[B13] Chen L. Tong Z. Luo H. Qu Y. Gu X. Si M. (2023). Titanium particles in peri-implantitis: distribution, pathogenesis and prospects. Int. J. Oral. Sci. 15, 49. doi: 10.1038/s41368-023-00256-x, PMID: 37996420 PMC10667540

[B14] Choi E. Y. Lim J.-H. Neuwirth A. Economopoulou M. Chatzigeorgiou A. Chung K.-J. . (2015). Developmental endothelial locus-1 is a homeostatic factor in the central nervous system limiting neuroinflammation and demyelination. Mol. Psychiatry 20, 880–888. doi: 10.1038/mp.2014.146, PMID: 25385367 PMC4351922

[B15] Chudnovskiy A. Pasqual G. Victora G. D. (2019). Studying interactions between dendritic cells and T cells *in vivo*. Curr. Opin. Immunol. 58, 24–30. doi: 10.1016/j.coi.2019.02.002, PMID: 30884422 PMC6927575

[B16] Chukkapalli S. S. Velsko I. M. Rivera-Kweh M. F. Larjava H. Lucas A. R. Kesavalu L. (2017). Global TLR2 and 4 deficiency in mice impacts bone resorption, inflammatory markers and atherosclerosis to polymicrobial infection. Mol. Oral. Microbiol. 32, 211–225. doi: 10.1111/omi.12165, PMID: 27224005 PMC5123973

[B17] Curtis M. A. Diaz P. I. Van Dyke T. E. (2020). The role of the microbiota in periodontal disease. Periodontol 83, 14–25. doi: 10.1111/prd.12296, PMID: 32385883

[B18] Diaz P. Gonzalo E. Villagra L. J. G. Miegimolle B. Suarez M. J. (2022). What is the prevalence of peri-implantitis? A systematic review and meta-analysis. BMC Oral. Health 22, 449. doi: 10.1186/s12903-022-02493-8, PMID: 36261829 PMC9583568

[B19] Eke P. I. Thornton-Evans G. O. Wei L. Borgnakke W. S. Dye B. A. Genco R. J. (2018). Periodontitis in US adults: national health and nutrition examination survey 2009-2014. J. Am. Dent. Assoc. 149, 576–588.e6. doi: 10.1016/j.adaj.2018.04.023, PMID: 29957185 PMC8094373

[B20] Ertugrul A. S. Sahin H. Dikilitas A. Alpaslan N. Bozoglan A. (2013). Comparison of CCL28, interleukin-8, interleukin-1β and tumor necrosis factor-alpha in subjects with gingivitis, chronic periodontitis and generalized aggressive periodontitis. J. Periodontal Res. 48, 44–51. doi: 10.1111/j.1600-0765.2012.01500.x, PMID: 22812409

[B21] Failer T. Amponsah-Offeh M. Neuwirth A. Kourtzelis I. Subramanian P. Mirtschink P. . (2022). Developmental endothelial locus-1 protects from hypertension-induced cardiovascular remodeling via immunomodulation. J. Clin. Invest. 132, e126155. doi: 10.1172/JCI126155, PMID: 35133978 PMC8920341

[B22] Ghensi P. Manghi P. Zolfo M. Armanini F. Pasolli E. Bolzan M. . (2020). Strong oral plaque microbiome signatures for dental implant diseases identified by strain-resolution metagenomics. NPJ Biofilms Microbiomes 6, 47. doi: 10.1038/s41522-020-00155-7, PMID: 33127901 PMC7603341

[B23] Giro G. Tebar A. Franco L. Racy D. Bastos M. F. Shibli J. A. (2021). Treg and TH17 link to immune response in individuals with peri-implantitis: a preliminary report. Clin. Oral. Investig. 25, 1291–1297. doi: 10.1007/s00784-020-03435-w, PMID: 32594309

[B24] Hajishengallis G. (2014). Immunomicrobial pathogenesis of periodontitis: keystones, pathobionts, and host response. Trends Immunol. 35, 3–11. doi: 10.1016/j.it.2013.09.001, PMID: 24269668 PMC3947349

[B25] Hajishengallis G. (2015). Periodontitis: from microbial immune subversion to systemic inflammation. Nat. Rev. Immunol. 15, 30–44. doi: 10.1038/nri3785, PMID: 25534621 PMC4276050

[B26] Hajishengallis G. Chavakis T. (2019). DEL-1-regulated immune plasticity and inflammatory disorders. Trends Mol. Med. 25, 444–459. doi: 10.1016/j.molmed.2019.02.010, PMID: 30885428 PMC6488420

[B27] Hajishengallis G. Darveau R. P. Curtis M. A. (2012). The keystone-pathogen hypothesis. Nat. Rev. Microbiol. 10, 717–725. doi: 10.1038/nrmicro2873, PMID: 22941505 PMC3498498

[B28] Hajishengallis G. Lamont R. J. (2012). Beyond the red complex and into more complexity: the polymicrobial synergy and dysbiosis (PSD) model of periodontal disease etiology. Mol. Oral. Microbiol. 27, 409–419. doi: 10.1111/j.2041-1014.2012.00663.x, PMID: 23134607 PMC3653317

[B29] Hajishengallis G. Lamont R. J. (2021). Polymicrobial communities in periodontal disease: Their quasi-organismal nature and dialogue with the host. Periodontol 86, 210–230. doi: 10.1111/prd.12371, PMID: 33690950 PMC8957750

[B30] Hasturk H. Kantarci A. Goguet-Surmenian E. Blackwood A. Andry C. Serhan C. N. . (2007). Resolvin E1 regulates inflammation at the cellular and tissue level and restores tissue homeostasis *in vivo*1. J. Immunol. 179, 7021–7029. doi: 10.4049/jimmunol.179.10.7021, PMID: 17982093

[B31] Hasturk H. Kantarci A. Ohira T. Arita M. Ebrahimi N. Chiang N. . (2006). RvE1 protects from local inflammation and osteoclastmediated bone destruction in periodontitis. FASEB J. 20, 401–403. doi: 10.1096/fj.05-4724fje, PMID: 16373400

[B32] Hasturk H. Schulte F. Martins M. Sherzai H. Floros C. Cugini M. . (2021). Safety and preliminary efficacy of a novel host-modulatory therapy for reducing gingival inflammation. Front. Immunol. 12. doi: 10.3389/fimmu.2021.704163, PMID: 34589083 PMC8475270

[B33] Huang M. Wang C. Li P. Lu H. Li A. Xu S. (2024). Role of immune dysregulation in peri-implantitis. Front. Immunol. 15. doi: 10.3389/fimmu.2024.1466417, PMID: 39555067 PMC11563827

[B34] Kampits C. Montenegro M. M. Ribeiro I. W. J. Furtado M. V. Polanczyk C. A. Rösing C. K. . (2016). Periodontal disease and inflammatory blood cytokines in patients with stable coronary artery disease. J. Appl. Oral. Sci. 24, 352–358. doi: 10.1590/1678-775720160082, PMID: 27556206 PMC4990364

[B35] Kensara A. Hefni E. Williams M. A. Saito H. Mongodin E. Masri R. (2021). Microbiological profile and human immune response associated with peri-implantitis: A systematic review. J. Prosthodont. 30, 210–234. doi: 10.1111/jopr.13270, PMID: 33016381

[B36] Kriebel K. Hieke C. Müller-Hilke B. Nakata M. Kreikemeyer B. (2018). Oral biofilms from symbiotic to pathogenic interactions and associated disease –connection of periodontitis and rheumatic arthritis by peptidylarginine deiminase. Front. Microbiol. 9. doi: 10.3389/fmicb.2018.00053, PMID: 29441048 PMC5797574

[B37] Lee C.-T. Li R. Zhu L. Tribble G. D. Zheng W. J. Ferguson B. . (2021). Subgingival microbiome and specialized pro-resolving lipid mediator pathway profiles are correlated in periodontal inflammation. Front. Immunol. 12. doi: 10.3389/fimmu.2021.691216, PMID: 34177951 PMC8222734

[B38] Lee C.-T. Teles R. Kantarci A. Chen T. McCafferty J. Starr J. R. . (2016). Resolvin E1 reverses experimental periodontitis and dysbiosis. J. Immunol. 197, 2796–2806. doi: 10.4049/jimmunol.1600859, PMID: 27543615 PMC5026932

[B39] Li S. Sun F. Wei Y. Nie Y. Wu X. Hu W. (2023). Mucosal bleeding correlates with submucosal microbial dysbiosis in peri-implant mucositis of patients with periodontitis. Clin. Oral. Implants Res. 34, 947–957. doi: 10.1111/clr.14120, PMID: 37358250

[B40] Lin D. Li L. Sun Y. Wang W. Wang X. Ye Y. . (2014). IL-17 regulates the expressions of RANKL and OPG in human periodontal ligament cells via TRAF6/TBK1-JNK/NF-κB pathways. Immunology. doi: 10.1111/imm.12395, PMID: 25263088 PMC4557684

[B41] Löe H. Theilade E. Jensen S. B. (1965). Experimental gingivitis in man. J. Periodontol. 36, 177–187. doi: 10.1902/jop.1965.36.3.177, PMID: 14296927

[B42] Loos B. G. Van Dyke T. E. (2020). The role of inflammation and genetics in periodontal disease. Periodontol 83, 26–39. doi: 10.1111/prd.12297, PMID: 32385877 PMC7319430

[B43] Maekawa T. Hosur K. Abe T. Kantarci A. Ziogas A. Wang B. . (2015). Antagonistic effects of IL-17 and D-resolvins on endothelial Del-1 expression through a GSK-3β-C/EBPβ pathway. Nat. Commun. 6, 8272. doi: 10.1038/ncomms9272, PMID: 26374165 PMC4573473

[B44] Mehrnia N. (2025a). Figure 1. Cycle of inflammation in periodontal and peri implant tissues. Available online at: https://app.biorender.com/illustrations/67f35bd3befd403f1c04c847 (Accessed April 7, 2025).

[B45] Mehrnia N. (2025b). Figure 2.Immune Response across periodontal lesion stages. Available online at: https://app.biorender.com/citation/692cf83a8c8ba68b1d4d4cf5 (Accessed November 30, 2025).

[B46] Meuric V. Le Gall-David S. Boyer E. Acuña-Amador L. Martin B. Fong S. B. . (2017). Signature of microbial dysbiosis in periodontitis. Appl. Environ. Microbiol. 83, e00462–e00417. doi: 10.1128/AEM.00462-17, PMID: 28476771 PMC5494626

[B47] Modafferi C. Grippaudo C. Corvaglia A. Cristi V. Amato M. Rigotti P. . (2025). Genetic testing in periodontitis: A narrative review on current applications, limitations, and future perspectives. Genes 16:1308. doi: 10.3390/genes16111308, PMID: 41300760 PMC12652003

[B48] Noronha Oliveira M. Schunemann W. V. H. Mathew M. T. Henriques B. Magini R. S. Teughels W. . (2018). Can degradation products released from dental implants affect peri-implant tissues? J. Periodontal Res. 53, 1–11. doi: 10.1111/jre.12479, PMID: 28766712

[B49] Page R. C. Schroeder H. E. (1976). Pathogenesis of inflammatory periodontal disease. A summary of current work. Lab. Investig. J. Tech. Methods Pathol. 34, 235–249., PMID: 765622

[B50] Pan W. Wang Q. Chen Q. (2019a). The cytokine network involved in the host immune response to periodontitis. Int. J. Oral. Sci. 11, 1–13. doi: 10.1038/s41368-019-0064-z, PMID: 31685798 PMC6828663

[B51] Pan W. Wang Q. Chen Q. (2019b). The cytokine network involved in the host immune response to periodontitis. Int. J. Oral. Sci. 11, 1–13. doi: 10.1038/s41368-019-0064-z, PMID: 31685798 PMC6828663

[B52] Preshaw P. M. Bissett S. M. (2019). Periodontitis and diabetes. Br. Dent. J. 227, 577–584. doi: 10.1038/s41415-019-0794-5, PMID: 31605062

[B53] Richter G. M. Schaefer A. S. (2025). Genetic susceptibility to periodontitis. J. Periodontal Res. doi: 10.1111/jre.70002, PMID: 40642787

[B54] Sahrmann P. Gilli F. Wiedemeier D. B. Attin T. Schmidlin P. R. Karygianni L. (2020). The microbiome of peri-implantitis: A systematic review and meta-analysis. Microorganisms 8, 661. doi: 10.3390/microorganisms8050661, PMID: 32369987 PMC7284896

[B55] Salminen A. Hyvärinen K. Ritari J. Caetano A. Kambur O. Mäntylä P. . (2025). Genetic loci associated with periodontitis: the finnGen study based on national health registers. J. Clin. Periodontol. 52, 1263–1275. doi: 10.1111/jcpe.14193, PMID: 40682483 PMC12377943

[B56] Scannapieco F. A. Dongari-Bagtzoglou A. (2021). Dysbiosis revisited: Understanding the role of the oral microbiome in the pathogenesis of gingivitis and periodontitis: A critical assessment. J. Periodontol. 92, 1071–1078. doi: 10.1002/JPER.21-0120, PMID: 33902163 PMC8380683

[B57] Sedghi L. M. Bacino M. Kapila Y. L. (2021). Periodontal disease: the good, the bad, and the unknown. Front. Cell. Infect. Microbiol. 11. doi: 10.3389/fcimb.2021.766944, PMID: 34950607 PMC8688827

[B58] Serhan C. N. Chiang N. Van Dyke T. E. (2008). Resolving inflammation: dual anti-inflammatory and pro-resolution lipid mediators. Nat. Rev. Immunol. 8, 349–361. doi: 10.1038/nri2294, PMID: 18437155 PMC2744593

[B59] Sirisereephap K. Maekawa T. Tamura H. Hiyoshi T. Domon H. Isono T. . (2022). Osteoimmunology in periodontitis: local proteins and compounds to alleviate periodontitis. Int. J. Mol. Sci. 23, 5540. doi: 10.3390/ijms23105540, PMID: 35628348 PMC9146968

[B60] Song B. Zhang Y. L. Chen L. J. Zhou T. Huang W. K. Zhou X. . (2017). The role of Toll-like receptors in periodontitis. Oral. Dis. 23, 168–180. doi: 10.1111/odi.12468, PMID: 26923115

[B61] Van Dyke T. E. (2017). Pro-resolving mediators in the regulation of periodontal disease. Mol. Aspects Med. 58, 21–36. doi: 10.1016/j.mam.2017.04.006, PMID: 28483532 PMC5660638

[B62] Van Dyke T. E. Baima G. Romandini M. (2025). Periodontitis: microbial dysbiosis, non-resolving inflammation, or both? J. Periodontal Res. doi: 10.1111/jre.13424, PMID: 40657987

[B63] Van Dyke T. E. Bartold P. M. Reynolds E. C. (2020). The nexus between periodontal inflammation and dysbiosis. Front. Immunol. 11. doi: 10.3389/fimmu.2020.00511, PMID: 32296429 PMC7136396

[B64] Van Dyke T. E. Serhan C. N. (2003). Resolution of inflammation: A new paradigm for the pathogenesis of periodontal diseases. J. Dent. Res. 82, 82–90. doi: 10.1177/154405910308200202, PMID: 12562878

[B65] Wang W. Wang X. Lu S. Lv H. Zhao T. Xie G. . (2021). Metabolic disturbance and th17/treg imbalance are associated with progression of gingivitis. Front. Immunol. 12. doi: 10.3389/fimmu.2021.670178, PMID: 34234776 PMC8257051

[B66] Wei X. Qian S. Yang Y. Mo J. (2024). Microbiome-based therapies for periodontitis and peri-implantitis. Oral. Dis. 30, 2838–2857. doi: 10.1111/odi.14782, PMID: 37890080

[B67] Zaric S. Shelburne C. Darveau R. Quinn D. J. Weldon S. Taggart C. C. . (2010). Impaired immune tolerance to Porphyromonas gingivalis lipopolysaccharide promotes neutrophil migration and decreased apoptosis. Infect. Immun. 78, 4151–4156. doi: 10.1128/IAI.00600-10, PMID: 20679442 PMC2950342

[B68] Zhao M. Zheng Z. Li C. Wan J. Wang M. (2022). Developmental endothelial locus-1 in cardiovascular and metabolic diseases: A promising biomarker and therapeutic target. Front. Immunol. 13. doi: 10.3389/fimmu.2022.1053175, PMID: 36518760 PMC9742254

